# Procedural and declarative memory in children with and without specific language impairment

**DOI:** 10.3109/13682820902752285

**Published:** 2009-12-10

**Authors:** Jarrad A. G. Lum, Celin Gelgic, Gina Conti-Ramsden

**Affiliations:** † Deakin University, Melbourne Australia; ‡ The University of Manchester, Manchester, United Kingdom

**Keywords:** specific language impairment (SLI), memory, language development, cognition

## Abstract

**Background:**

Much evidence has accumulated to indicate memory deficits in children with specific language impairment. However, most research has focused on working memory impairments in these children. Less is known about the functioning of other memory systems in this population.

**Aims:**

This study examined procedural and declarative memory in young children with and without specific language impairment.

**Methods & Procedures:**

A total of 15 children with specific language impairment and 15 non-impaired children of comparable age, gender and handedness were presented with measures of procedural and declarative memory. Procedural memory was assessed using a Serial Reaction Time (SRT) Task in which children implicitly learnt a ten-item sequence pattern. Declarative memory for verbal and visual information was assessed using paired associative learning tasks.

**Outcomes & Results:**

The results from the SRT Task showed the children with specific language impairment did not learn the sequence at levels comparable with the non-impaired children. On the measures of declarative memory, differences between the groups were observed on the verbal but not the visual task. The differences on the verbal declarative memory task were found after statistically controlling for differences in vocabulary and phonological short-term memory.

**Conclusions & Implications:**

The results were interpreted to suggest an uneven profile of memory functioning in specific language impairment. On measures of declarative memory, specific language impairment appears to be associated with difficulties learning verbal information. At the same time, procedural memory is also appears to be impaired. Collectively, this study indicates multiple memory impairments in specific language impairment.

**What this paper adds**

It is known that young people with specific language impairment can have difficulties with memory, in particular working memory. What is less known is how other memory systems function in children with specific language impairment. The present study found that children with specific language impairment appear to have memory deficits that involve both the procedural memory system and the declarative memory system. Interestingly, deficits in declarative memory appear to be confined to the verbal domain whilst the evidence for procedural memory deficits suggests a broader deficit.

## Introduction

Children who meet the diagnostic criteria for specific language impairment (SLI) have considerable difficulty with the acquisition and use of language in the absence of intellectual impairments, sensory loss or central nervous system disease (DSM-IV-TR; American Psychiatric Association (APA) 2000; ICD-10; World Health Organization (WHO) 1994). In addition to language impairments, these children typically perform poorer on number of tasks assessing cognitive functioning (for example, Gathercole and Baddeley 1990; Miller *et al*. 2001; and Tallal *et al*. 1981). To date a substantial number of studies indicate that memory functioning may be impaired in SLI (for example, Archibald and Gathercole 2006; Fazio 1996; and Marton and Schwartz 2003). This has led to one view that memory limitations may underlie some of the language difficulties experienced by these children (for example, Montgomery 2003). At the same time it has also been noted that these memory problems may be secondary to language difficulties (for example, Weismer and Edwards 2006). In understanding the relationship between memory and language functioning in SLI, most studies have focused on working memory (for example, Archibald and Gathercole 2006). However, very little research has been undertaken to examine other memory systems in this population and their relationship to language. This study examined procedural and declarative memory in children with SLI.

Research undertaken with clinical and non-clinical populations, as well as human and non-human animals, suggests the presence of multiple memory systems which can be differentiated functionally and neuro-anatomically (for example, Squire 2004; and Tulving 1985). One taxonomy distinguishes between declarative and procedural memory (Squire *et al*. 1993). A key process completed by the declarative memory system is to bind different or arbitrarily related representations or perceptual experiences (Mayes *et al*. 2007; Squire *et al*. 2004). The procedural memory system is involved in the acquisition and retrieval of habits, motor and cognitive skills (Knowlton *et al*. 1994, 1996; Nissen and Bullemer 1987; Seger 2006). Unlike declarative memory, encoding and retrieval via the procedural system can occur without awareness (for example, Cohen and Squire 1980). Procedural learning typically occurs through repeated exposure to a stimulus or repeating a motor sequence. As a consequence it seems that this systems appears better suited to encoding and retrieving information which has a sequential or probabilistic structure (Knowlton *et al*. 1994). The acquisition of new motor skills is typically cited to demonstrate learning that is supported by procedural memory. For example, learning to use a manual transmission in a car involves numerous repetitions of sequenced motor processes. Initially, awareness in executing motor processes is required; however, with repeated practice deployment of the skill can be achieved implicitly.

The acquisition of language seemingly requires both procedural and declarative systems (Bates 2004). For instance, young infants are able to learn and store statistical regularities about the phonology of incoming speech (for example, Saffran *et al*. 1996). This process appears congruent with learning supported by the procedural memory system. Declarative memory can be considered important in supporting word learning since this process is dependent upon, in part, learning form and meaning relationships (Naigles 2003). One theory which attempts to account for the roles of multiple memory systems in language learning has been forwarded by Ullman (2001, 2004) in the Declarative/Procedural Model of Language. According to the model, declarative and procedural memory support different components of language. Declarative memory is argued to be involved in learning and storing lexical items. This is because the binding of conceptual, phonological and semantic representations is a process which is carried out by this memory system. Procedural memory is considered to support the acquisition and storage of grammatical forms which are seemingly rule based such as the English regular past tense (Ullman *et al*. 1997). This claim is made on the basis that the procedural memory system is well suited to learning and storing regularities. As a final note, irregular verb forms are proposed to be acquired and stored by the declarative memory system given the arbitrary relationship between the word form and its referent. Ullman and Pierpont (2005) extended the Declarative/Procedural Model to account for the language difficulties reported SLI. There is substantial evidence suggesting that English speaking children with SLI have considerable difficulty with the acquisition of grammar relative to other components of language (Leonard 1997). The regular past tense appears particularly problematic for these individuals (for example, Rice *et al*. 1998). To account for this profile Ullman and Pierpont (2005) proposed that grammatical impairment in SLI might arise from an impaired procedural memory system. At the same time, given that lexical knowledge appears relatively less impaired, it suggested that declarative memory is intact in these individuals.

At present little is known about procedural and declarative memory functioning in SLI. One study known to the authors has examined procedural memory in SLI. Tomblin *et al*. (2007) investigated procedural learning in adolescents with and without SLI. The study group consisted of 38 adolescents with SLI (mean age = 15 years) and the control group consisted of 47 non-language impaired individuals (mean age = 14.76 years), matched on non-verbal IQ. Procedural learning was assessed using a Serial Reaction Time (SRT) Task. SRT Tasks have been used extensively to study this memory system and much evidence has accumulated supporting their validity (Freed *et al*. 1989; Knopman and Nissen 1991; Nissen 1992; Siegert *et al*. 2006). In the SRT Task used by Tomblin *et al*. (2007) participants were asked to press one of four buttons on a response pad which corresponded to one of four locations that a visual stimulus could appear on a computer screen. The only instruction provided was to press the correct button on the response pad after the visual stimulus had appeared. Participants were presented with four blocks of visual stimulus presentations. Each block consisted of 100 trials. On the first and fourth block the stimulus appeared randomly. However, on the second and third blocks presentation of the visual stimulus followed a ten-item sequence. Procedural learning was evaluated by examining the reaction times. On the blocks which contained the sequenced stimulus presentations, the non-impaired children showed an initial decline in reaction times then reached a plateau. This pattern of results suggests procedural learning has occurred. In contrast, the reaction times of the children with SLI decreased at a slower rate. The slower learning rates were interpreted to suggest a procedural learning impairment in SLI.

Little is also known about declarative memory functioning in SLI. Assessing the associative functioning aspects of the declarative memory system typically involve asking participants to learn associations between unrelated visual or verbal information over a number of trials (Baron 2004). Bavin *et al*. (2005) presented a visual associative learning task to five-year-old children with and without SLI. On this task children were seated in front of a computer screen. A target visual stimulus briefly appeared in the middle of the screen. Surrounding the visual stimulus were several boxes which then revealed their contents one at a time. The target stimulus was located in one of the boxes. After the contents of all boxes had been revealed, children were asked to point to the box which contained the target stimulus seen earlier. As children progress through the task they are presented with an increasing number of visual stimuli. Overall, both groups of children were able to learn to associate an equal number of visual stimuli to their locations in the boxes. However, Bavin *et al*. did report that the children with SLI required more stimulus presentations in order to learn the location of the target stimulus in comparison to the controls.

Whether children with SLI can associate information for verbal information is yet to be thoroughly investigated. In one of the validation studies undertaken with the Children's Memory Scale (Cohen 1997) children with and without SLI were tested on a word pairs learning task. On this task children were presented with a list of semantically unrelated word pairs. The list of word pairs was presented three times. Following each presentation children were provided with the first word of the pair and asked to recall the second. At the conclusion of the task children were asked to recall both word pairs. The children with SLI recalled significantly fewer words than the non-impaired children. This result would suggest the presence of an impairment associated with declarative memory for verbal information. However, one potential confound with this interpretation follows Gathercole *et al*.'s (1997) study demonstrating an association between performance on a word pairs task and vocabulary in non-impaired children. In accounting for this finding Gathercole *et al*. suggested children with larger vocabularies might be more capable of learning pairs because ‘they have greater opportunity to discover semantic associations between unrelated pairs of words’ (p. 976). From this perspective, the reported difficulty children with SLI have with learning word pairs may reflect smaller vocabularies rather than an impairment with declarative memory. Another issue not be overlooked is that children with SLI have been shown to have impairments with the short-term storage of phonological information (for example, Gathercole and Baddeley 1990). This might also contribute to the difficulty children with SLI have with learning semantically unrelated word pairs. This is because in learning to associate two words, children need to store the first word temporarily until the second has been presented.

Individual studies indicate mixed findings concerning declarative and procedural memory functioning in SLI. While there is some research suggesting procedural memory is impaired in SLI the status of other memory systems is not yet clear. In particular, interpreting past research on word pair learning in SLI is complicated by potential group differences in vocabulary and phonological short-term memory. A final concern is that the *profile* of declarative and procedural memory functioning in SLI is not yet clear. A key prediction forwarded from the Declarative/Procedural Model of SLI (Ullman and Pierpont 2005) is that impairments in procedural memory are observed in the presence of normal declarative memory functioning. However, empirically evaluating this claim on the basis of past research is problematic at present given that than individual studies typically focus on a single memory system. In light of the aforementioned issues, the current study evaluated both declarative and procedural memory functioning in a single sample of children with and without SLI. Two hypotheses were forwarded based on Ullman and Pierpont's (2005) model of SLI. First, significant differences between the groups were expected on the measure of procedural memory. Second, no significant differences were predicted on the measure of declarative memory.

## Method

### Participants

Fifteen children with SLI (eight male, seven female) and 15 typically developing (TD) children (eight male, seven female) of comparable age, gender and handedness participated in this study. The age of the children ranged from 7;3 to 8;4 years. Children were recruited from primary schools located in Melbourne, Australia. None of the participants in this study were reported to have sensory impairments or behavioural problems as reported by teachers and parents.

### Identification of children with and without SLI

Children with and without SLI were identified using standardized tests of language and non-verbal reasoning. In addition to psychometric testing all children with SLI were receiving in-school support in relation to a language/learning problem. Children's language skills were assessed using the Clinical Evaluation of Language Fundamentals 4th Edition — Australian (Semel *et al*. 2003). This test produces several indices which describe language functioning. The Core Language Score (CLS) provides an overall summary of children's expressive and receptive language skills. The Receptive Language Index (RLI) and Expressive Language Index (ELI) provide a measure of receptive and expressive language skills respectively. All core and index scores are standardized to a mean of 100 and standard deviation (SD) of 15. Children with SLI were identified on the basis of a CLS of less than 85. This cut-off score has been shown to produce the highest sensitivity and specificity levels in an Australian sample of children. Semel *et al*. (2003) demonstrated that that a cut-off score of 85 or less is associated with a sensitivity rate of 0.83 and a specificity rate of 0.90. All children with SLI obtained a CLS of less than 85. The children in the TD group obtained CLS between 90 and 110.

Non-verbal reasoning skills were assessed using the Raven's Coloured Progressive Matrices (Raven 1998). This test assesses children's non-verbal reasoning skills and has positive correlations with the Performance IQ (*r* = 0.52), Verbal IQ (*r* = 0.54) and Full IQ (*r* = 0.55) from the Wechsler Intelligence Scale for Children (Chalmers and Frederick 1955). Scores on RCPM were standardized to a mean of 100 and an SD of 15. All but two of the children with SLI obtained standardized RCPM scores between 85 and 115. These two children obtained standardized scores of 118 and 125. To match these scores two children without SLI with RCPM standardized scores of 117 and 125 were included in the sample.

Children were also screened for handedness using the Quantification of Hand Preference (Bishop *et al*. 1996). The need to control for handedness in this study was necessary because the task used to measure procedural learning required participants to respond using their right hand. Subsequently, handedness may contribute to the between-subject variance observed on this task. On the QHP task participants are seated in front of seven sets of three cards at 30° increments measured from the midline. Each set of cards is placed 40 cm from participants. The cards displayed pictures of common objects (for example, chair, house, and rabbit). During testing children were asked to pick up the cards in a random order and the hand used to pick up the card was noted by the test administrator (for details, see Bishop *et al*. 1996). Following Calvert and Bishop (1998), the dependent variable obtained from this task is the proportion of cards picked up with the right hand.

Descriptive statistics for the SLI and TD groups' age and results from the tests are presented in Table 1. Differences between groups were evaluated with independent samples *t*-tests and an effect size measure *r*^2^. In Table 1, *r*^2^ represents the proportion of variance accounted in the dependent variable (for example, age, CLS, and ELI) by the independent variable (group membership). Table 1 shows statistically significant differences between the groups on all measures from the CELF-4 Australian. Groups differed maximally on CLS scores which was to be expected given this variable was used to identify whether children were SLI. The groups were comparable with respect to age and handedness with group membership accounting less than 1% of variance in these variables. This was also expected given that groups were matched on these variables. RCPM scores approached statistical significance with group membership accounting for 11.1% of variance. To ensure that potential group differences on the memory tasks did not reflect differences in RCPM, scores, this measure was used as a covariate in all analyses.

**Table 1:** Descriptive statistics for age and tests by group

	SLI (*n* = 15)				TD (*n* = 15)				Comparisons		
			
	Mean	Standard deviation	Minimum	Maximum	Mean	Standard deviation	Minimum	Maximum	*t*	*p*	*r*^2^
Age^a^	84.73	10.98	67	100	84.07	9.51	67	99	0.178	0.86	0.001
CLS^b^	72.67	12.79	41	85	103.80	12.79	96	114	8.860	< 0.001	0.729
ELI^b^	73.40	13.49	47	89	107.73	13.49	100	118	4.128	< 0.001	0.751
RLI^b^	78.20	12.08	57	100	94.60	12.08	70	111	9.191	< 0.001	0.378
RCPM^b^	102.47	10.66	85	125	108.80	7.66	97	125	1.869	0.072	0.111
QHP^d^	0.81	0.28	0.19	1.00	0.79	0.27	0.05	1.00	0.344^f^	0.733	0.004

a Notes: Months.

b Test standardized to a mean of 100 and standard deviation of 15.

c Maximum score = 40.

d Proportion of reaches with the right hand.

e Proportion of correct responses.

f Arcsine transformation was applied to the data before analysis to correct for non-normality.

CLS, Core Language Score; ELI, Expressive Language Index; RLI, Receptive Language Index; RCPM, Raven's Coloured Progressive Matrices; QHP, Quantification of Hand Preference.

### Materials

Children were presented with a battery of tasks which assessed procedural and declarative memory. Because performance on these tasks may also be influenced by motor speed, handedness, phonological short-term memory or vocabulary (depending on the task), several additional tasks were also presented to the children. The data obtained from these tasks were used as covariates in the analyses examining memory. Each task is now described.

#### Measure of procedural learning

**Figure 1:**
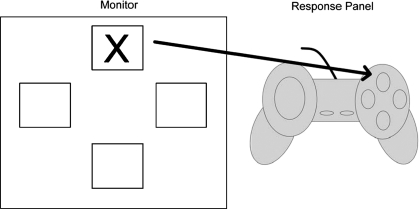
Schematic overview of the SRT Task. Children were asked to press the button on the response pad that matched the location of a visual stimulus which appeared on a computer screen.

Procedural learning was examined using a variation of Nissen and Bullemer's (1987) Serial Reaction Time (SRT) Task. On the SRT Task participants were seated approximately 40 cm in front of a laptop computer and presented with a Gravis Gamepad Pro which was attached to the computer. The Gravis Gamepad Pro consists of four buttons arranged in the shape of a diamond, which children operated using their right thumb. Using the Gravis Gamepad Pro enabled us to present the SRT Task to children as a computer game. Maintaining children's interest in the SRT Task was important given the duration of the task. The spatial locations that the visual stimulus could appear were marked by four boxes with white boarders. The arrangement of these boxes was ordered in a diamond configuration which matched the location of the buttons on the response pad. The white boxes subtended 6.4 × 6.4 degrees of visual angle. A schematic overview of the task is presented in Figure 1.

During testing children were instructed that a smiley face would appear inside one of the four boxes and their task was to press one of the buttons on the response pad that matched the visual stimulus' location (Figure 1). Participants were given ten practise trials to ensure they understood the instructions. Children were then informed that the real game would to start. The test items were then presented. This part of the task consisted of 90 stimulus presentations divided into five blocks. Unbeknown to children on Blocks 1–4 presentation of the visual stimulus followed a ten-item sequenced pattern which was the same used by Nissen and Bullemer (1987). This was: 4, 2, 3, 1, 3, 2, 4, 3, 2 and 1 where the left most positioned box on the computer monitor is labelled as ‘one’, the upper-most box is ‘two’, and so on. On the fifth block the visual stimulus appeared in a pseudo-random order. On this block the visual stimulus appeared randomly on the screen with the following two constraints. First, the visual stimulus appeared in each box on the computer screen an equal number of times as for Blocks 1–4. Second, the probability of the visual stimulus appearing in one of the four spatial locations, given the location of the preceding location was kept the same as for Blocks 1–4. That is, in the repeating sequence, if the visual stimulus appeared in Location 1, there was a 50% chance that on the next trial it would appear in Locations 3 or 4. If the stimulus appeared in Location 2, there was a 33.3% chance the stimulus would appear in either Location 1, 3, or 4. If, the stimulus appeared in Location 3, there was a 33.3% chance it would appear in Location 1 and a 66.7% chance it would appear in Location 2. Finally, if the visual stimulus appeared in Location 4, there was a 50% chance that on the next trial the stimulus would appear in either Locations 2 and 3. Constructing the random block in this manner controlled for the possibility that any differences between non-random and random blocks reflected that children had only learnt associations between single transitions (Jackson *et al*. 1995).

Children's accuracy and reaction times (RTs) were obtained from the SRT Task. Accuracy on this task was quantified by computing the proportion of correct button presses for each child. That is, whether the child pressed the correct button on the response pad following the appearance of the visual stimuli in one of the four spatial locations. RTs were measured from the appearance of the visual stimulus to the time children pressed a button on the response pad. For each child the median RTs for each block was computed. Following past research (for example, Siegert *et al*. 2006) procedural memory on this task was quantified by subtracting RT observed in the fourth block from those in the fifth block. Using this method procedural learning occurs if positive values are obtained. That is, RTs are higher in the random block (Block 5) in comparison to Block 4. This is because if the children had not learnt anything about the pattern then RT should continue to decrease in the random block or reach asymptote. Individuals with procedural memory impairments show a smaller difference in RT between random and sequenced blocks (for example, Siegert *et al*. 2006).

At the conclusion of the task children's explicit knowledge of the pattern was tested. This was undertaken by informing children that the visual stimulus followed a pattern. Following this all children were presented with four explicit recall trials. On each trial the visual stimulus was presented in one of the four spatial locations and children were asked where they thought it would appear next up to a maximum of ten locations. None of the children participating in the study was able to recall the ten-item sequence pattern.

#### Measures of declarative memory for verbal and visual information

Declarative memory for verbal and visual information was tested in this study. Declarative memory for verbal information was assessed using the word pairs subtests from the Children's Memory Scale (WPCMS; Cohen 1997). On the WP-CMS the children were asked to learn a single list of ten semantically unrelated word pairs (for example, listen-magic) across three trials. Learning lists of word pairs has been shown to be impaired in individuals with pathology associated with the left temporal lobe. This is one of the key neural structures that supports declarative memory (for example, Wood *et al*. 2000). On the WP-CMS the children were orally presented with a list of ten word pairs. Children were then given the first word of each pair (for example, listen) and asked to recall the second (for example, magic). The same procedure was followed for the second and third trial, although the order word-pairs were presented differed between trials. An immediate recall trial was presented following the third trial. On this trial participants were asked to recall both pairs of words. Children's performance on this test was summarized by the Word Pairs Total Score. This is the sum of the correct responses the child made across the three presentation trials and number of correct word-pairs recalled on the immediate recall trials. The highest score that can be obtained from this task was 40.

Declarative memory for visual information was assessed using the Paired Associates Learning (PAL) subtest from the Cambridge Automated Neuropsychological Test Battery (Cambridge Cognition Ltd. 2006). This subtest is reported to be sensitive to right temporal lobe functioning (Cambridge Cognition Ltd. 2006). In addition to this, impairments with declarative memory for non-verbal information have been reported in individuals with epilepsy with neural pathology found in the right temporal lobe (Jambaqué *et al*. 2007). On the PAL subtest participants were seated in front of a touch screen. The task commenced with a target pattern briefly appearing in the centre of the screen. Surrounding the pattern were boxes which briefly display their contents on at a time. The target pattern appeared in only one of these boxes. Children were asked to tap the box which contained the target pattern. The difficulty of the task increases as children progress through the stages. On Stages 1–2, children are asked to learn the location of one pattern. On Stages 3–4 there are two patterns, on Stages 5–6 there are three patterns, on Stage 7 there are six patterns and on Stage 8 there are eight patterns. On each stage children try to learn associations between the target pattern and its correct location in one of the surrounding boxes before proceeding to the next stage. If an incorrect response is made, that is children point to a box which does not contain the location of the target pattern, then the target pattern reappears briefly in the centre of the screen. If children are unable to learn the location of the pattern within ten repeat trials the task stops. Performance on this task was described with two variables; total number of completed stages and the total number of errors.

### Controlling for potential confounds on tasks assessing procedural and declarative memory

#### Measures of motor speed

Differences in motor speed between SLI and TD groups may lead to differences on the SRT Task. Subsequently, it was necessary to present participants with independent measures of motor speed which were to be used as covariates in the analyses. In this study children were presented with (1) Motor Screening Test (MOT) from the CANTAB and (2) a Tapping Task previously used by Bishop (2002). On the MOT children are seated in front of a touch screen computer. A computer graphic shaped as an ‘X’ appears on the screen. Using their index finger, children are asked to tap the graphic. There are a total of three practice and ten trials on this task. Two dependent variables were obtained from this task. The first is the average response latency. That is the difference in time from when the ‘X’ graphic appeared on the screen and when children tapped the touch screen computed from the test trials. The second dependent variable described children's accuracy at tapping the ‘X’ graphic. This was measured as the mean distance from the middle of the ‘X’ to the point on the screen touched by the child across the ten test trials. The distance is measured in pixel units given a screen resolution of 640 × 350 pixels.

Bishop's (2002) tapping task was also presented to participants. In this task children are presented with a counter. On each trial children hold the counter in either their left or right hand and asked to press the counter button as many times in 30 seconds as possible. There are a total of four trials on this task with each thumb assessed with two trials. For this study the total number of presses was computed separately for the left and right. This task was selected because of its similarity with the movement required on the SRT Task.

#### Measure of phonological short-term memory

Phonological short-term memory may explain potential differences between SLI and TD groups on the WP-CMS task. In order to control for this confound children were presented with the Children's Test of Nonword Repetition (Gathercole and Baddeley 1996). This test comprises 40 non-words which vary from two to five syllables. In this study non-words were presented to children via headphones and children's responses were recorded and scored for accuracy offline.

#### Measure of vocabulary

Differences in vocabulary may also represent another confounding variable on the WP-CMS task (Gathercole *et al*. 1997). Controlling for vocabulary was achieved by presenting children with Form M of the Peabody Picture Vocabulary Test — Revised (Dunn and Dunn 1981). Data from this test were used as a covariate in some of the analyses. The PPVT-R is a standardized measure of receptive vocabulary. On this task children were asked to point to one of four pictures which matched an orally presented target word. Raw scores from this test were used as a covariate in the analyses. The maximum score on this test was 175.

### Procedure

The battery of tests was presented individually and in a quiet room located in the children's respective schools. The battery was presented over three 30–40-minute sessions. There was an approximately seven-day break between testing sessions. Presentation of the tasks was counterbalanced in order to average potential differential carry-over effects.

## Results

The analyses examined whether the SLI and TD group differed on the SRT Task (which measured procedural learning) and on the WP-CMS and PAL tasks (which measured declarative memory). The data from the SRT Task are presented first. The proportion of correct responses on this task is presented in Table 2. All the children in the TD group performed well above chance level (that is, obtained at accuracy rate of greater than 25%). All but one child in the SLI group consistently performed above chance level across the five blocks. This child's mean accuracy across the blocks ranged from 0.26 to 0.30. These data were subsequently excluded from the analyses of SRT data. Children's accuracy on the task was examined using a 2 (Group: SLI, TD) × 5 (Block: 1, 2, 3, 4 and 5) mixed design factorial analysis of variance (ANOVA). Preliminary data analysis revealed the scores were negatively skewed and an arcsine transformation was applied to the data. The results of this analysis indicated a significant main effect for Group (*F*(1, 27) = 8.398, *p* = 0.007, partial η^2^ = 0.237) and Block (*F*(2.829, 76.831)1 = 5.200, *p* = 0.001, partial η^2^ = 0.161). The interaction between Group and Block was not statistically significant (*F*(2.829, 76.831)2 = 1.299, *p* = 0.275, partial η^2^ = 0.046). The main effect found for Group indicates that the TD children were significantly more accurate than the children with SLI across all blocks. *Post-hoc* tests using the Bonferroni method were also undertaken to examine differences in accuracy between the five blocks. This analysis indicated a single significant difference, with accuracy on Block 5 being significantly lower than Block 2 (*p* = 0.012). No other comparisons were found to be statistically significant. To control for difference in overall accuracy between groups as well as within blocks only RTs associated with a correct response were used in the following analyses.

**Table 2:** Proportion of correct responses from the SRT Task

	SLI (*n* = 14)				TD (*n* = 15)			
		
	Mean	Standard deviation	Minimum	Maximum	Mean	Standard deviation	Minimum	Maximum
Block 1	0.85	0.20	0.40	1.00	0.97	0.04	0.89	1.00
Block 2	0.88	0.13	0.64	1.00	0.97	0.03	0.91	1.00
Block 3	0.89	0.10	0.61	1.00	0.95	0.04	0.87	1.00
Block 4	0.83	0.18	0.36	0.99	0.94	0.04	0.86	1.00
Block 5 (Random)	0.80	0.16	0.49	0.99	0.95	0.03	0.90	1.00

The second set of analyses examined the RT data from the SRT Task. Before undertaking these analyses the influence of general motor response times, as measured by the MOT subtest and Tapping Task, were considered. Descriptive statistics for these two tasks reported by group are presented in Table 3. Independent samples *t*-tests showed no significant differences between the groups on the motor tasks with group membership accounting for less than 1% of the variance. Thus the two groups appear comparable with respect to the independent measures of motor response speeds.

**Table 3:** Descriptive statistics for motor tasks by group

	SLI				TD				Comparisons		
			
	Mean	Standard deviation	Minimum	Maximum	Mean	Standard deviation	Minimum	Maximum	*t*	*p*	*r*^2^
MOT (Latency)^a^	1034.86	247.48	661	1433	1044.08	191.03	824	1494	0.113	0.911	< 0.001
MOT (Accuracy)^b^	11.28	1.90	8	16	10.75	2.60	6	15	0.616	0.543	0.014
Tapping task (left hand)^c^	140.58	16.61	108	161	139.07	15.22	113	168	0.641	0.798	0.002
Tapping task (right hand)^c^	167.67	29.30	128	210	165.80	28.09	105	199	0.470	0.861	0.001

a Notes: Milliseconds.

b Measured in pixel units on a screen with a 640 × 350 pixel resolution.

c Total number of button presses summed over two trials.

**Figure 2:**
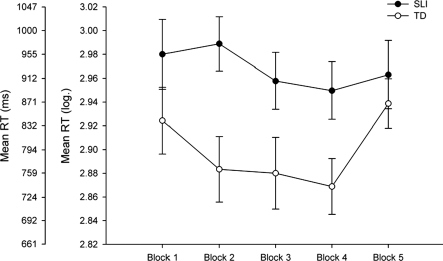
Mean (log-transformed) RTs from the SRT Task across the five block by group. Error bars show the standard error.

Children's RTs across the five blocks are presented in Figure 2. These data were transformed using a logarithmic transformation to correct for non-normality. Figure 2 shows that the children with SLI were slower than TD children at responding to the visual stimulus. Overall, for both groups there was a decrease in RT from Block 1 to Block 4. There is an increase in RT on presentation of the random block, although, it is noted that the increase is larger for the TD than for the SLI group. The key comparison of interest was whether the magnitude of difference between the fourth (final sequenced block) and fifth block (random block) differed between the groups. This method has been used to examine procedural learning in clinical and non-clinical groups (for example, Siegert *et al*. 2006). For the SLI group the mean difference in RT between fourth and fifth blocks was found to be 72 ms (SD = 52.41, minimum = −7.50; maximum = 189). The mean difference in RT for the controls was found to be 128.233 (SD = 63.75, minimum = −22.50, maximum = 222). Before analysing these data it was decided to control statistically for children's performance on the two measures of motor speed and RCPM scores. Even though no significant differences were observed on the MOT or Tapping Task between the groups, we wanted to examine procedural learning independently of motor speed and intelligence. Statistically controlling for these variables was achieved by using regression analyses. Specifically, RTs for each of the blocks from the SRT Task were regressed onto Tapping Task and MOT RTs as well as RCPM scores and the standardized residuals were saved. These standardized residuals were used in the analyses. This procedure removes the shared variance between each of the variables. Statistically controlling for differences using this approach is advantageous because avoids losing degrees of freedom had the data been analysed using an analysis of covariance (ANCOVA). Analysis of these standardized residuals indicated the magnitude of difference between the fourth and fifth Blocks was significantly larger for the TD than SLI group (*t*(27) = 2.545, *p* = 0.017, *r*^2^ = 0.193).

Data from the declarative memory tasks are now considered. Table 2 shows descriptive statistics from the WP-CMS and PAL by group. On the WP-CMS the SLI group recalled fewer words than the TD group. On the PAL all children in both groups completed all eight stages. However, children with SLI made more errors on the task. The data from the WP-CMS were analysed first using an independent samples *t*-test. Before analysing these data, differences between the groups on the CNRep and PPVT-R were considered. On the CNRep the TD obtained correctly repeated more non-words than the children with SLI (SLI = mean = 19, SD = 9; TD = mean = 33, SD = 5). This difference was statistically significant (*t*(28) = 2.721, *p* = 0.011, *r*^2^ = 0.209). Results from the PPVT-R also revealed the controls obtained a higher raw score than the children with SLI (SLI = mean = 74.27, SD = 13.59; TD = mean = 83.80, SD = 16.38), however, this difference was not found to be statistically significant (*t*(28) = 1.736, *p* = 0.094, *r*^2^ = 0.097). Power analysis indicated only a 38.8% change of detecting a statistically significant effect size of 0.097. Subsequently, it was decided to control for differences in PPVT-R as well as CNRep and RCPM scores. Statistically controlling for CNRep, RCPM and PPVT-R scores was achieved by using regression analyses and saving standardized residuals as described previously. An independent samples *t*-test was used to evaluate differences between the groups on adjusted WP-CMS scores. The results of this analysis revealed that the TD group recalled more items than the children with SLI (*t*(28) = 3.941, *p* < 0.001, *r*^2^ = 0.356).

The data from the PAL was analysed next. Table 4 shows that all children in both groups completed the eight stages. Given that both groups performed at ceiling on this dependent measure, inferential statistics could not be used to analyse the data. However, Table 2 shows that the children with SLI tended to make more errors on this task than controls. An analysis controlling for group differences in RCPM scores indicated the difference in errors on the PAL was not statistically significant (*t*(28) = 0.767, *p* = 0.449, *r*^2^ = 0.020).

**Table 4:** Descriptive statistics from WP-CMS and PAL by group

	SLI				TD			
		
	Mean	Standard deviation	Minimum	Maximum	Mean	Standard deviation	Minimum	Maximum
PAL (completed stages)^a^	8	0	8	8	8	0	8	8
PAL (number of errors)	17.67	11.93	1	44	12.00	9.19	1	24
WP-CMS^b^	6.27	5.08	0	17	16.67	5.25	8	24

a Notes: Maximum number of stages = 8.

b Maximum score = 40.

## Discussion

This study examined procedural and declarative memory in children with and without SLI. Two findings emerged from this study. First, consistent with Ullman and Pierpont's (2005) hypothesis the data from the SRT Task provided support for a procedural learning deficit in SLI. In one analysis the size of difference between the fourth and fifth block was found to be significantly larger in the TD than SLI group. This result was observed even though the two groups of children did not differ on the two independent measures assessing basic motor speed. In other SRT research this level of evidence has been sufficient in demonstrating a procedural memory deficit (for example, Siegert *et al*. 2006). Second, the results suggest an uneven declarative memory profile in SLI. Consistent with Ullman and Pierpont's model non-significant differences between SLI and TD children were observed on a visual declarative memory task. However, differences between groups were observed on a verbal declarative memory task. This result was not consistent with the Declarative/Procedural Model. Collectively, the results suggest procedural and declarative memory systems may be impaired in SLI.

The results from the SRT Task are consistent with those reported by Tomblin *et al*. (2007). In their study children with SLI were reported to have a procedural learning impairment based on the observation that the learning rates of the children with SLI were slower. In the current study procedural learning was examined using a version of Nissen and Bullemer's (1987) SRT Task. Our primary motivation for including this task was to allow more direct comparisons with populations known to have impairments with procedural memory. In previous research using similar versions of the SRT Task, individuals with Parkinson's and Huntington's disease (for example, Siegert *et al*. 2006) have been reported to smaller difference in RTs between blocks containing random and sequenced trials than controls. In the current study comparisons between the two groups of children mirrored these findings to some extent. In one analysis we reported a significant difference in implicit learning on the SRT Task between children with and without SLI. This result was observed after removing the variance associated with children's motor speed. It is also important to note that differences in handedness did not seem to be an appropriate explanatory variable since group membership accounted for less than 1% of the variance on the QHP. From this perspective the results reported in this study can generally be interpreted to suggest a procedural learning impairment in SLI.

The results from the declarative memory tasks revealed an uneven level of functioning which appeared to depend on whether the stimulus to be learnt was visual or verbal. The children with and without SLI performed at comparable levels on the PAL task. Both groups learnt to associate comparable numbers of visual stimulus to spatial locations. This results was comparable with those reported by Bavin *et al*. (2005) and suggests declarative memory for visual information may be intact in SLI. At the same time the children with SLI learnt fewer semantically unrelated word pairs than the non-impaired children. This result can be interpreted to suggest a declarative memory impairment for verbal information. These findings are comparable with those of Cohen (1997) who found children with SLI learnt fewer word pairs than controls. The current study extends these findings by replicating this result even after controlling for group differences in phonological short-term memory and vocabulary. Thus it is suggested that the difficulty children with SLI have with declarative memory for verbal information extends beyond their phonological short-term memory and vocabulary limitations.

A large number of studies have investigated memory functioning in SLI. The results of the current study along with those undertaken elsewhere indicate that multiple memory systems may be impaired in SLI. Specifically, it seems that working, procedural and verbal declarative memory are all affected in these children. At the same time this study does indicate sparing of declarative memory for visual information. Accounting for this pattern of findings is problematic at present. One possibility is that the language impairments are causally related to the memory problems in SLI (for a discussion, see Weismer and Edwards 2006). This proposal would be consistent with the difficulty children with SLI had with the word pairs task and not with the visual paired associates. However, not to be overlooked is that the children with SLI perform more poorly on the task of procedural memory which is a measure of implicit motor skill learning. Resolving this question is dependent upon further research into the role of procedural memory in language acquisition and functioning.

Another outstanding question arising from this study concerns the relationship between memory systems. One possible interpretation of the data is that all memory problems found in children with SLI might be secondary to working memory impairments. This is possible since information processed by the declarative and procedural memory systems are initially short-term representations which might include working memory. Resolving this question will require further research. At present much research has been undertaken examining the interactions between memory systems in non-clinical adult populations. For instance, it has been shown that procedural and declarative memory systems may either compete or cooperate during learning (for example, Gold 2004). Elsewhere it has been shown that working memory may moderate the relationship between these two systems (Foerde *et al*. 2006). These findings indicate that impairment in a single memory system will have flow on effects for others. Resolving this issue in SLI will require assessing multiple memory systems in this population.

## Conclusion

This study found that children with specific language impairment (SLI) performed more poorly than non-impaired children on tests of procedural memory and a test of declarative memory for verbal information. Interestingly, both groups were comparable on a declarative memory task involving non-meaningful visual stimuli. When considered along with previous research (for example, Archibald and Gathercole 2006), the results from the present study suggest multiple memory systems may be impaired in SLI. For both working and declarative memory there appears to be an uneven profile of functioning with group differences being more apparent for verbal than visual information. At the same time it does not appear that all non-verbal memory systems are intact in SLI. The procedural memory task used in this study involved implicitly learning a visual sequence. The children with SLI did not learn this pattern with equal proficiency as the non-impaired children. Thus procedural memory in children with SLI does not appear to be functioning at comparable levels with non-impaired children. An important outstanding question from this study concerns the relationship between memory functioning and language impairment in SLI. Future research is required to address the relationship between memory and language functioning in SLI. Nevertheless, the results of this study do highlight that it may be insufficient to interpret language impairments in SLI solely in terms of impaired working memory functioning. In order to understand fully the relationship between memory and language functioning in SLI it will be necessary to consider multiple memory systems.
